# Vibration Serviceability of Footbridges Made of the Sustainable and Eco Structural Material: Glued-Laminated Wood

**DOI:** 10.3390/ma15041529

**Published:** 2022-02-18

**Authors:** Paweł Hawryszków, Jan Biliszczuk

**Affiliations:** Faculty of Civil Engineering, Wrocław University of Science and Technology, Wybrzeże Wyspiańskiego 27, 50-370 Wrocław, Poland; jan.biliszczuk@pwr.edu.pl

**Keywords:** footbridges, dynamic behaviour, glued-laminated wood

## Abstract

In this paper, dynamic analyses of two untypical, modern footbridges made of glued-laminated timber are presented. One of them is among the longest cable-stayed bridges for pedestrians in the world, made of such a structural material. Both structures are qualified as having low sensitivity to vibrations. The results of numerical modal analysis using FEM and non-destructive experimental dynamic tests of investigated footbridges are compared. Important differences in obtained results are captured, which are identified as the positive effect in relation to design aspects. Moreover, the same in situ measurements confirm the high level of damping in footbridges made of glued-laminated wood, which is a very significant and distinguishing feature not commonly recognized. The study also calls attention to the choice of timber as an advisable material for footbridges. This is not only because of environmentally friendly and aesthetic reasons, but also due to providing highly satisfying vibration comfort for pedestrians.

## 1. Introduction

One of the basic scientific problems of contemporary bridge engineering, in relation to pedestrian footbridges, is dynamic sensitivity. Modern footbridges are more sensitive to vibrations. This is caused by technologically advanced materials used for their construction, which have better strength parameters compared to those used before, along with a tendency for designing atypical and original structures. Landmark structures should be attractive in terms of an interesting architectural shape, which quite often contradicts the classic principles of designing footbridges. Apart from the progress in material technology, a factor which makes it possible to meet engineering challenges is the development of computer software, which solves complex design problems. A consequence of the mentioned factors is the greater sensitivity of modern structures to dynamic loads [1,2,3,4,5,6,7,8,9].

Nowadays, many different structural materials are used to construct footbridges—mainly steel and concrete [10,11,12]. Wood [13,14] is not so popular, although it can be characterized by a lot of positive, important aspects, e.g., sustainability, ecology, or renewability. In the times of carbon footprint reduction and a green world tendency [15], wood should be significantly considered as a suitable and modern material.

The glued-laminated wood is also friendly material to pedestrians. The structures built with its application are generally aesthetic [13,16].

Finally, in the context of this study, a positive potential of wooden structures in terms of dynamics should be highlighted. It is caused by the damping ratio [17], which has the highest value among structural materials (see Section 4). This feature is very important in relation to the dynamic behaviour of wooden footbridges.

In this paper, two glued-laminated timber pedestrian bridges built in Poland are presented. Both footbridges are characterized by unusual structural solutions, modern construction material, and low sensitivity to vibrations. The results of the dynamic tests and the design aspects of both structures are further described.

## 2. Materials and Structures

Glued-laminated wood [18] combines traditional, natural, and biologically renewable material with the modern technology of production. The fabrication process consists of preparing wooden boards and their permanent connection by using specialized glue. The advantage of such technology is that there is no limit in the length or height of the wooden girders, and no deformation of the timber beams due to rheological processes. Such wooden girders are also better protected against biological corrosion thanks to deep impregnation of the boards.

**Figure 1:**
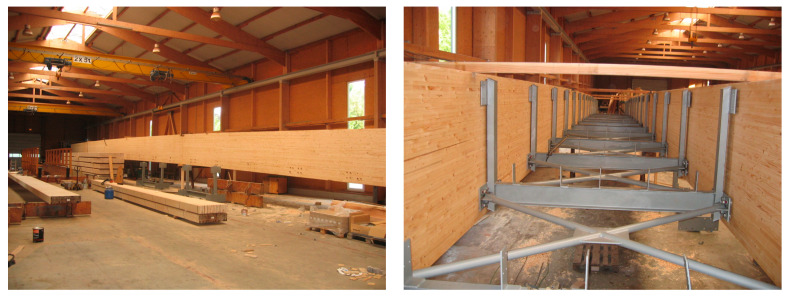
Environmental-friendly glued-laminated wood used for construction of the footbridge’s main girders and the deck (Photo credit: Schmees & Lühn Holz- und Stahlingenieurbau GmbH & Co. KG and Mosty-Wrocław Design & Research Office).

The prefabrication of glued-laminated wooden girders and the footbridge deck during construction are presented in Figure 1. The photo was taken in the woodworking company in Germany during the deck’s pre-assembly. The footbridge with detailed innovative structural solutions is described in Section 2.1.

### 2.1. Cable-Stayed Structure

**Figure 2:**
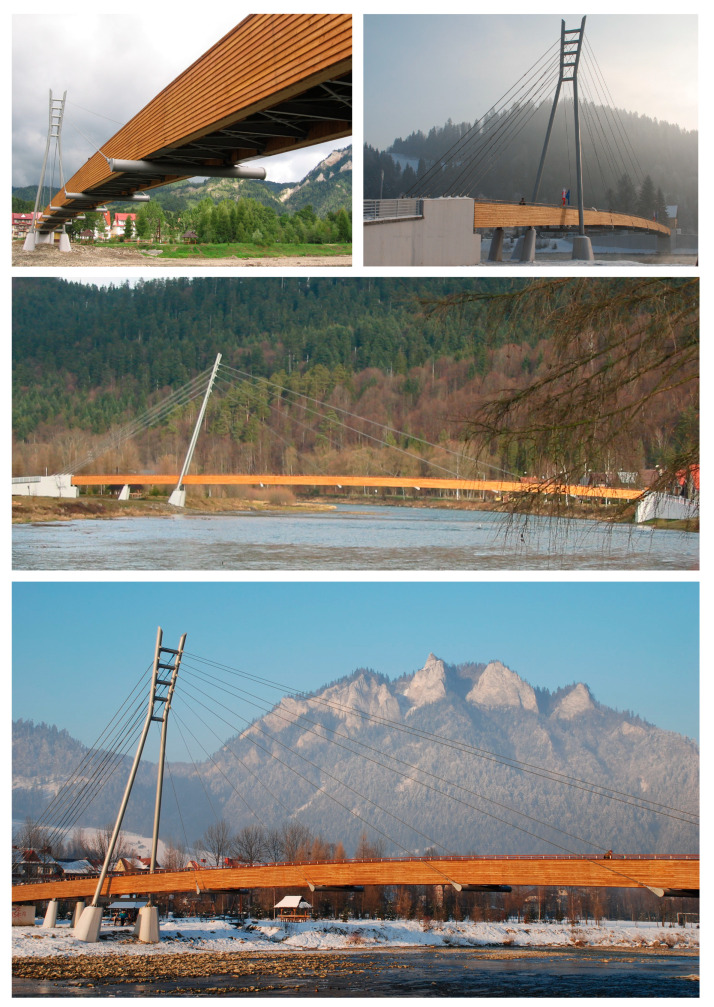
The cable-stayed footbridge in the Pieniny mountains made of glued-laminated wood.

The footbridge [19,20] was built over the Dunajec River in a mountain resort of the Pieniny National Park and connects two countries, Poland and Slovakia. The structure has significant influence on this attractive border region through the development and improvement of the tourist infrastructure. The ambition was to create a footbridge as a landmark structure, corresponding with the surrounding landscape (Figure 2).

**Figure 3:**
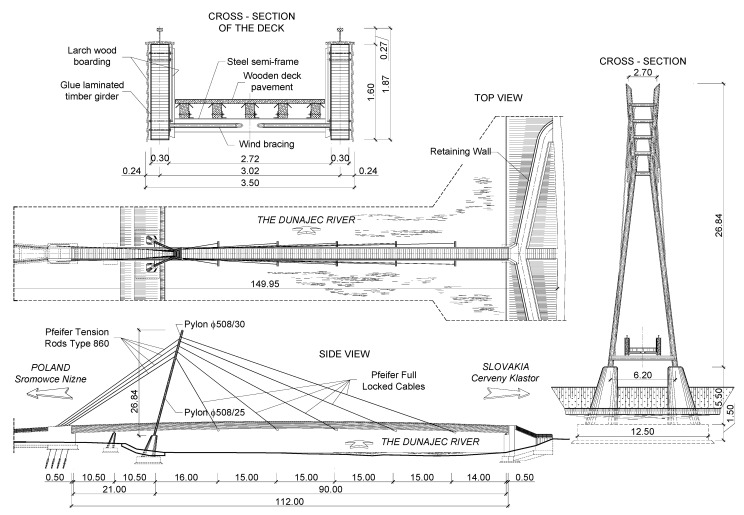
Structural solutions of the footbridge in the Pieniny mountains.

The footbridge was designed as a cable-stayed structure with a deck made of glued-laminated wood. The main span is 90.0 m long, whereas the side spans are each 10.50 m long. The total length of the structure is 149.95 m (Figure 3).

**Figure 4:**
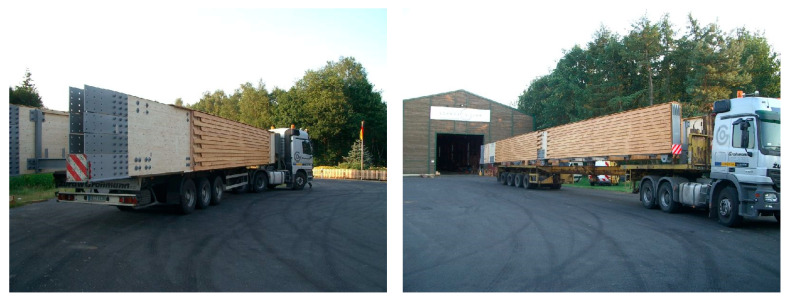
Assembling segments of the deck during their transport on the specialized lorries from the woodworking company in Germany to the construction site in Poland (Photos credit: Schmees & Lühn Holz- und Stahlingenieurbau GmbH & Co. KG and Mosty-Wrocław Design & Research Office).

The deck of the footbridge was constructed with wood and steel elements. The width of the deck is 2.50 m, and the total width is 3.50 m. The total height of the deck is 1.87 m. The deck consists of two main girders made of glued-laminated wood braced by steel semi-frames and wind bracing. The girders were designed using pinewood of GL32 class with a rectangular cross-section of 1.60 m × 0.30 m. The total length of the wooden girders is 112.0 m. They are protected against atmospheric and biological corrosion by additional, external wooden layers. The deck was made of prefabricated segments 12.2 m + 5 × 15.0 m + 24.80 m long (Figure 4). The assembling joints were constructed from steel screwed sheets and are discussed in detail in Section 3.1.

**Figure 5:**
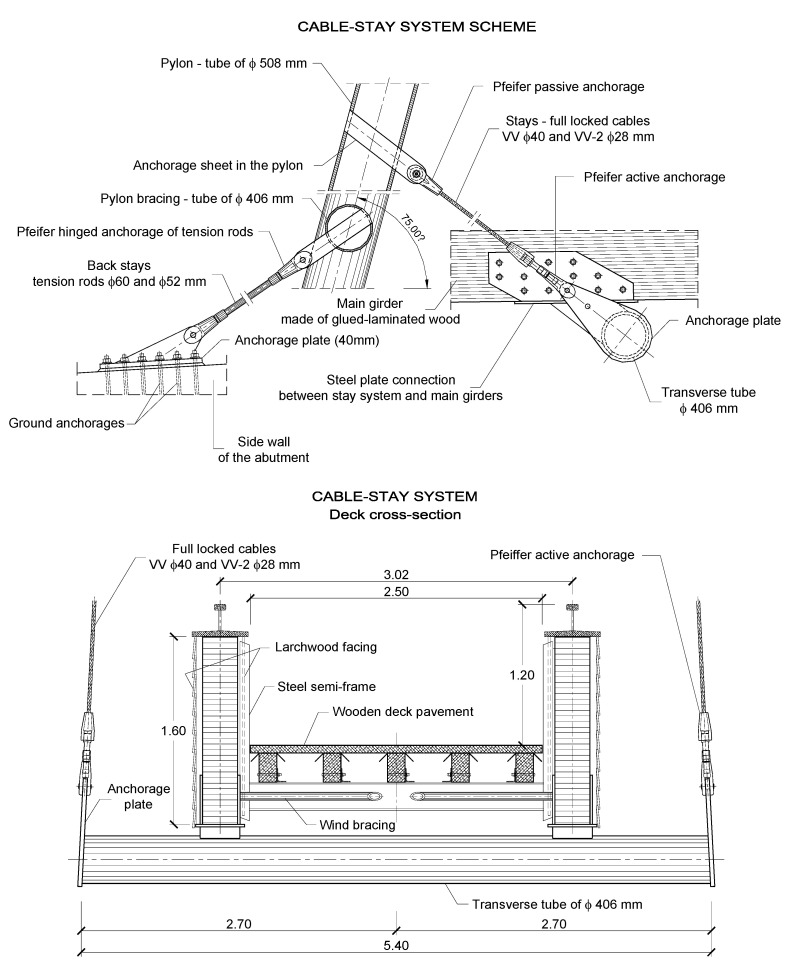
The stay cable system of the footbridge in the Pieniny mountains.

The wooden deck is supported by five pairs of stays in a distance of 15.0 m. The stay cable system was manufactured by Pfeifer (Figure 5). Full locked cables of Ø40 mm or Ø28 mm were used in the main span and tensioned rods type 860 of Ø60 mm or Ø52 mm were used for the back-stays. The pylon was constructed from steel tubes with a diameter of 508 mm and was inclined in the direction of the main span. The height of the pylon is 26.84 m above the concrete support.

### 2.2. Beam Structure

**Figure 6:**
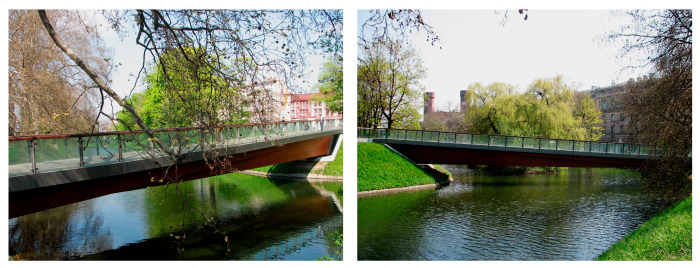
The beam footbridge in Wrocław made of glued-laminated wood.

**Figure 7:**
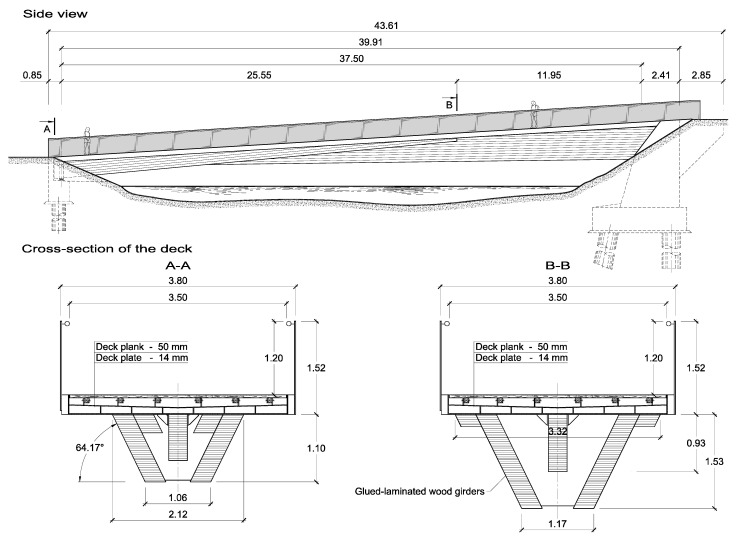
Structural solutions of the footbridge in Wrocław.

The footbridge [21] was designed in the south-west part of the Old Town in Wrocław (Figure 6). The structure (Figure 7) connects the busy junction with a quiet walk area. Heavy traffic on the streets and a lot of trees planted along the moat are characteristic for that location. There is a promenade, where pedestrians can rest in the shadow of the trees. Bicycle paths were designed there, along with a small square with a round flowerbed on the moat’s bend. This green area is only about 0.5 km far from the Town Hall of Wrocław. It is situated in the city centre and belongs to the monumental section, so it is often visited by both office workers and tourists. Its construction improved pedestrian traffic in this zone. The surroundings of the footbridge and characteristics of its location are shown in Figure 6.

Design conditions forced the footbridge to be built to about 40 m in length. Its width has been specified to be 3.50 m. A difference in ground level between both of the moat’s banks caused the longitudinal slope of the footbridge to be about 6%. The footbridge was designed for pedestrians, as well as cyclists.

The footbridge was designed as a single span beam structure with the span of 37.50 m long (see Figure 7). The total length of the structure is 43.61 m, and the width of the pavement on the deck is 3.50 m. The main girders were made of glued-laminated wood, class GL32. They have variable cross-section. The height changes in a range of 0.20 m ÷ 2.10 m, while the width is constant at 0.30 m. Both side girders lean about 30 degrees from the vertical. The main girders are braced by wooden crossbeams and tension rods.

The deck was designed in the form of a closed box. The deck’s elements (deck plate, longitudinal ribs, and crossbeams) were designed to be made of plates of different thickness. The deck pavement is made of 5.0 cm thick boards supported on six longitudinal wooden beams of 80 mm × 80 mm cross-section.

On both sides of the moat, concrete abutments were designed. The structure is fixed to one abutment, whereas on the opposite side it is supported on two bearings. This structural solution caused different dimensions of both abutments (see Figure 7).

## 3. Results

### 3.1. Dynamic Analysis of the Cable-Stayed Structure

**Figure 8:**
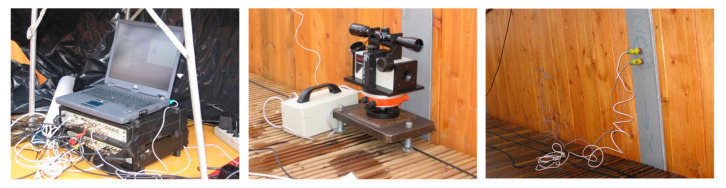
Measurement devices (Bruel & Kjaer amplifiers, Noptel OY PSM200 laser device and Endevco accelerometers).

In the experiments, ten accelerometers and a laser device were used (Figure 8). It was sufficient to register the dynamic response of the footbridge in the vertical and horizontal directions, and then restore the modal shapes corresponding to the lowest natural frequencies.

Five measurement points were located on both wooden main beams. At each point, one piezoelectric accelerometer of the 7752-1000 Endevco type was installed. The 3650C PULSE system produced by Bruel & Kjaer Sound & Vibration Measurement (Virum, Denmark) was used to measure and analyse the vibrations (Figure 8).

**Figure 9:**
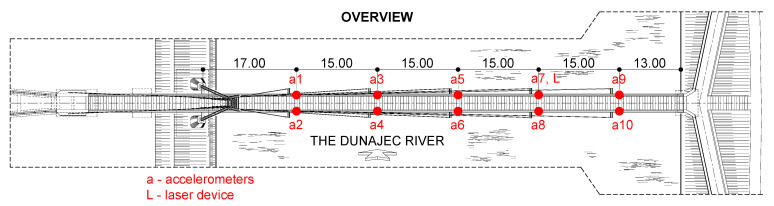
Location of measurement devices.

One measurement point was doubled by the NOPTEL OY PSM200 laser device to record the vibrations in displacement of the selected point of the structure. The laser device consisted of a laser transmitter (placed on an undeformable place outside the footbridge) and a receiver (placed on the footbridge). The location of all measurement devices is presented in Figure 9.

**Figure 10:**
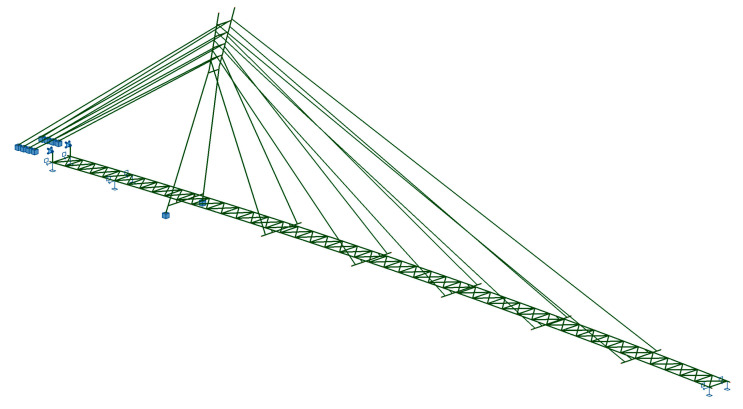
Numerical model of the structure.

A three-dimensional numerical model of the footbridges was used for dynamic calculations (Figure 10). The FEM model consisted of 455 bar elements and 251 nodes. In the case of some structural elements, an offset function was applied to model a proper location of element nodes. The boundary conditions of the model are presented in Figure 10.

**Figure 11:**
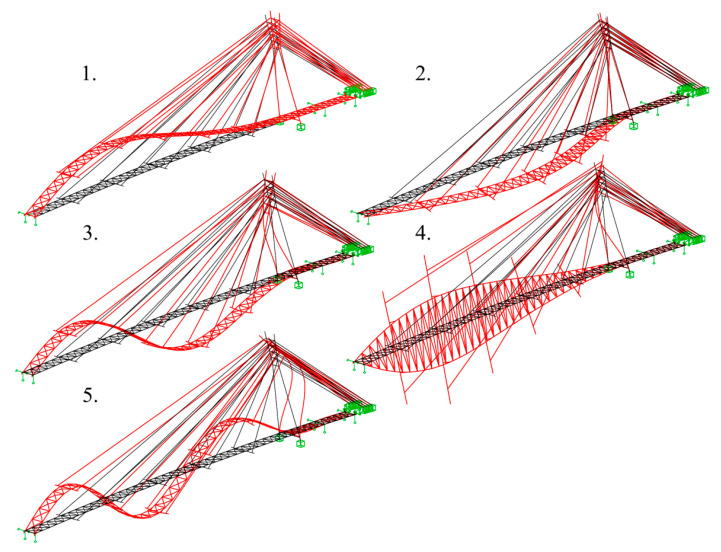
Modal shapes: 1. f_1_^V^ = 1.35 Hz (vertical), 2. f_1_^H^ = 1.41 Hz (horizontal), 3. f_2_^V^ = 2.47 Hz (vertical), 4. f_1_^T^ = 2.89 Hz (torsional), 5. f_3_^V^ = 4.18 Hz (vertical).

The main verification of the deck’s dynamic properties was aimed at the determination of the modal shapes and natural frequencies. The results of the modal analysis carried out on the FEM model of the structure are presented in Figure 11.

The research programme consisted of normal live loads and vandal actions to the footbridge. The normal live loads test examined the influence of various kinds of pedestrian activity on the footbridge’s behaviour. The programme involved the group of 12 pedestrians walking, jogging, or fast running. The live loads simulated regular pedestrian traffic on the bridge, and the dynamic response under such conditions was measured. Vandal type excitation consisted of synchronized walking or running and rhythmical half-crouching at the antinodes of respective modes (according to the results of the computational modal analysis). A metronome was used to determine the rate of crouching or path rate. The main aim of the vandal live loads was to check the structure’s safety and behaviour in the extreme dynamic conditions.

**Figure 12:**
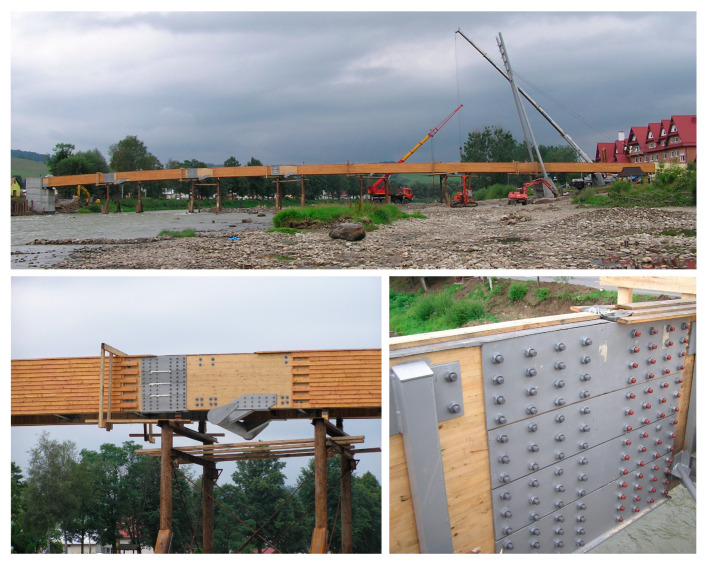
Installation of the deck on the temporary supports and assembling joints in the wooden girders.

**Figure 13:**
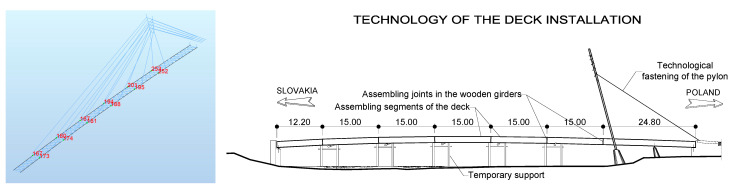
Location of assembling joints.

The values of the identified natural frequencies by using Fourier Transform were lower than the calculated ones (see Table 1 and Figure 11). This indicates smaller stiffness of the structure, which could result from the assembling joints in the wooden girders. The main beam segments (Figure 4) are connected with steel plates and screws (Figure 12), as a result of the construction technology (Figure 13). These joints probably decreased the stiffness of the structure compared to the computational model’s stiffness. In the computations, the main beams were reflected as continuous beams. This hypothesis has been verified below.

**Figure 14:**
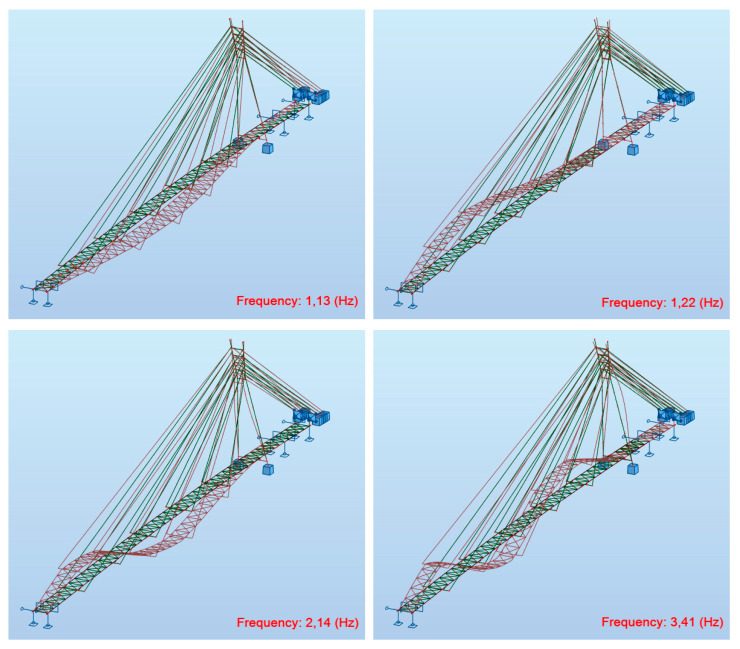
Results of dynamic calculations conducted on the updated model.

The FEM model has been updated with the modified joint stiffness to verify the theory regarding differences in frequency results. The assembling joints were defined based on the design and technology of the deck installation. The location of assembling joints is presented in Figure 13, whereas results of dynamic calculations conducted on the updated model are shown in Figure 14. A greater accuracy of the updated model was achieved in terms of all analysed modal shapes. The comparison of frequencies between the experiment and the updated FEM model is presented in Table 2. The differences between the in situ measurements and the calculated measurements are negligible. Moreover, a very interesting fact was discovered regarding the direction of vibrations in connection with changing the order of modal shapes (Table 3). In the case of the original FEM model, the vertical mode was before the horizontal mode. In the case of the updated FEM model, the order is consistent with the experiment—the horizontal mode is the first one, with the lowest value of frequency.

**Figure 15:**
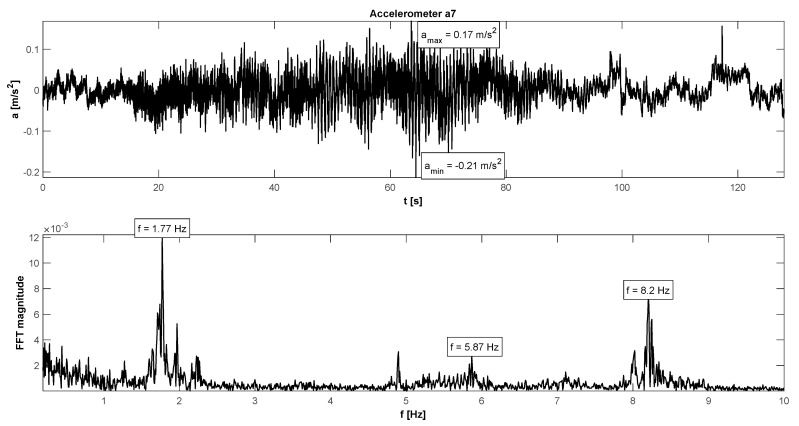
Signal of vertical vibrations in the time and frequency domain registered for 12 people walking.

**Figure 16:**
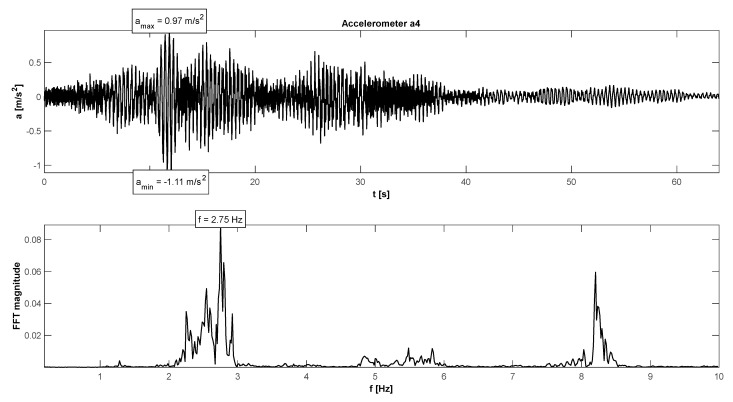
Signal of vertical vibrations in the time and frequency domain registered for 12 people running.

**Figure 17:**
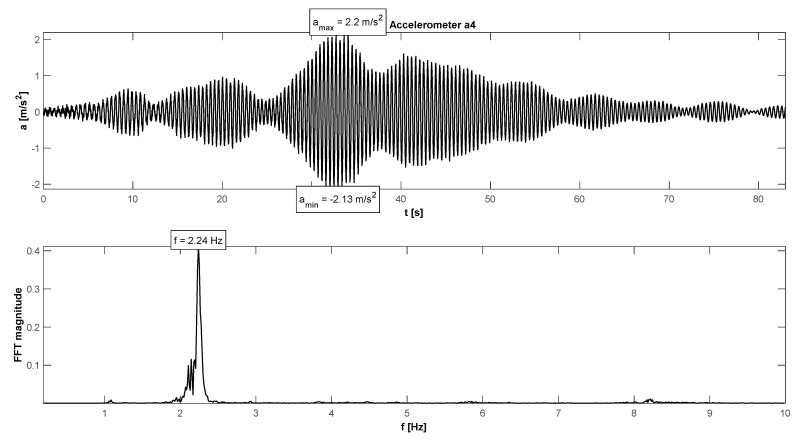
Signal of vertical vibrations in the time and frequency domain registered for the synchronized walking of 12 people.

**Figure 18:**
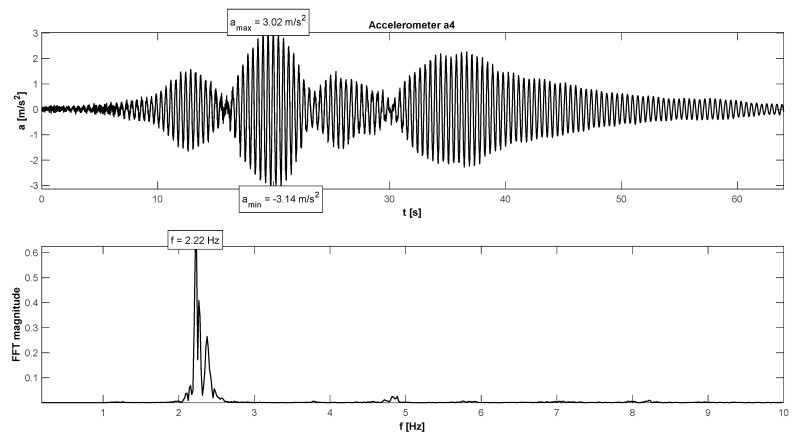
Signal of vertical vibrations in the time and frequency domain registered for the synchronized running of 12 people.

**Figure 19:**
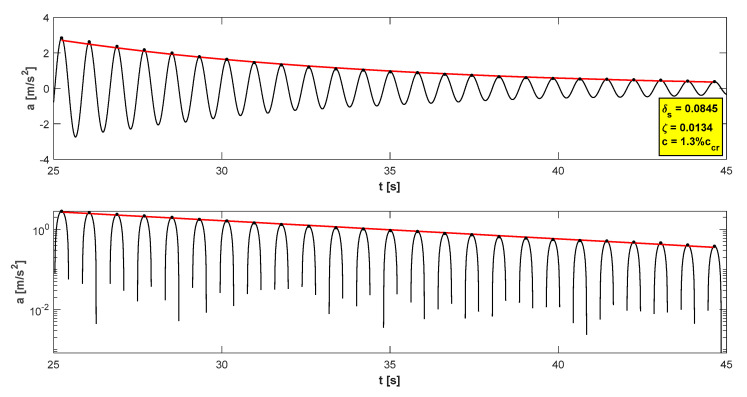
Determination of the damping coefficient.

The in situ tests have proven satisfactory behaviour in the footbridge under normal service conditions—accelerations did not exceed the admissible limits (see Table 4). The maximum accelerations were 0.21 m/s^2^ in walking conditions (Figure 15), 1.11 m/s^2^ in jogging conditions (Figure 16), and 1.38 m/s^2^ in fast running conditions. Synchronization of pedestrian activity caused a higher dynamic response of the structure, e.g., up to 2.20 m/s^2^ in synchronized walking conditions (Figure 17), 3.14 m/s^2^ in synchronized jogging conditions (Figure 18), and 4.19 m/s^2^ at some vandal excitations, such as half-crouching. The comfort limit of acceleration is usually defined as 0.50 m/s^2^ ÷ 0.70 m/s^2^ in the literature for walking pedestrians [22,23,24,25,26,27,28,29]. However, for running people, whose sensitivity to vibrations is lower, the comfort limit may be higher. According to the research of Hawryszków, it may be defined as 1.50 m/s^2^ [30]. In all cases of the normal activity of pedestrians, the comfort limit was fulfilled. In the case of vandal excitations, only the safety of the structure should be guaranteed (the human comfort criteria may be exceeded). The damping ratio determined by using the Logarithmic Decrement Method was above average and equals to 1.3% (Figure 19).

### 3.2. Dynamic Analysis of the Beam Structure

**Figure 20:**
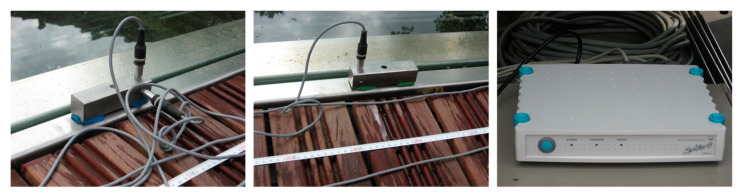
Measurement devices (HBM GmbH accelerometers and amplifier).

**Figure 21:**
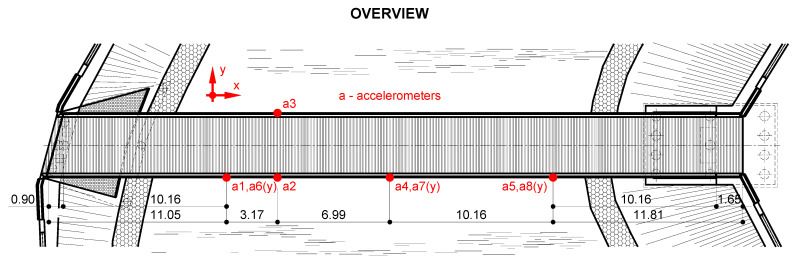
Location of the measurement devices.

The measurement devices were installed in four cross-sections of the footbridge. In the experiments, eight accelerometers of Hottinger Baldwin Messtechnik GmbH (Darmstadt, Germany), B12/200 type were used (Figure 20). The vibrations in both directions (vertical and horizontal) were measured. The location of the measurement devices is presented in Figure 21.

**Figure 22:**
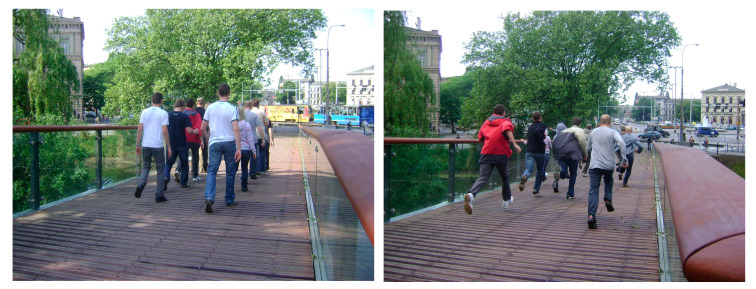
Examples of the dynamic tests carried out on the footbridge in Wrocław (synchronized walking and running).

A similar research programme was applied with the same load schemes as in the case of the footbridge in the Pieniny mountains. Ten pedestrians took part in dynamic tests of the footbridge in Wrocław (Figure 22).

**Figure 23:**
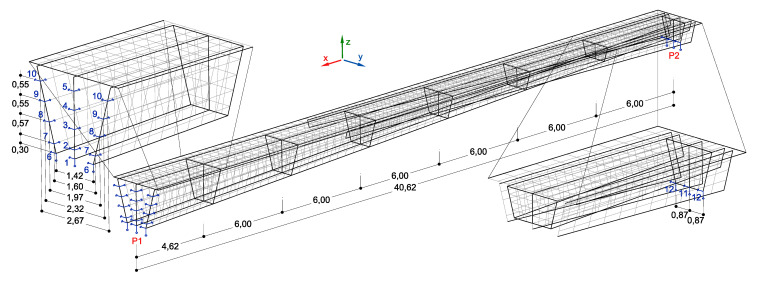
Numerical model of the structure [31].

A three-dimensional numerical model of the footbridge was used for dynamic calculations (Figure 23). The FEM model consisted of shell elements only, for which material and geometrical characteristics were defined to properly model the complicated cross-section and unusual shape of the structure. The boundary conditions of the model are presented in Figure 23.

**Figure 24:**
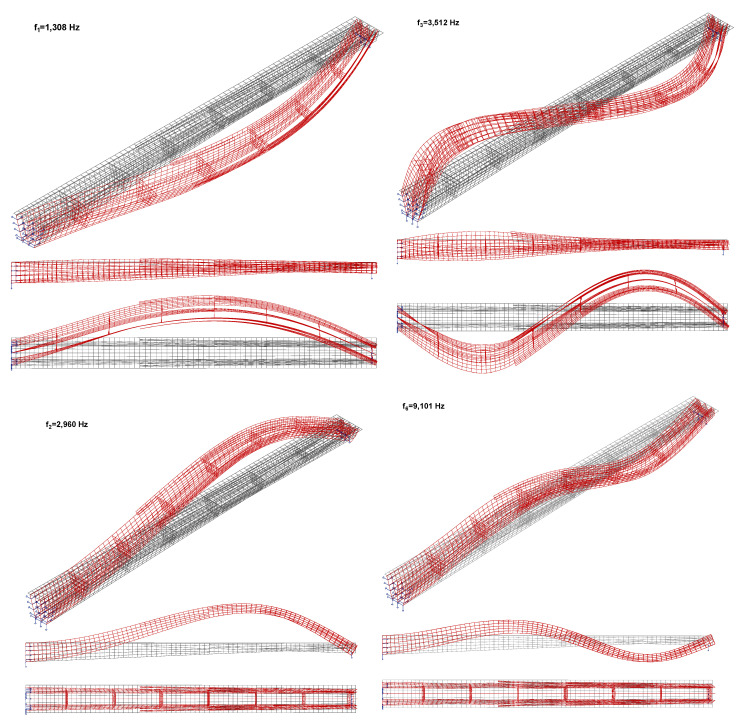
Modal shapes: f_1_^H^ = 1.31 Hz, f_2_^H^ = 3.51 Hz (horizontal) and f_1_^V^ = 2.96 Hz, f_2_^V^ = 9.10 Hz (vertical) [32].

The results of the modal analysis carried out on the FEM model of the structure are presented in terms of identified natural frequencies in Table 5, and in terms of modal shapes of corresponding frequencies in Figure 24. The Fast Fourier Transform (FFT) was used to identify frequencies. The accuracy of the model according to the first bending mode of vibrations was high. In the case of horizontal vibrations, greater stiffness of the structure was determined. The higher than expected differences between measured and calculated results mainly concern the horizontal direction, and are most likely connected with the very unusual shape of the structure and the complicated 3D shell-elements of the FEM model. As this work mainly concerns experimental campaigns (supported by FEM model analysis) and is focused on vertical vibrations as they are usually connected with the comfort of pedestrians crossing the footbridge, the problem of inaccuracy in results of natural frequencies has not been further analysed and can be part of a future study.

**Figure 25:**
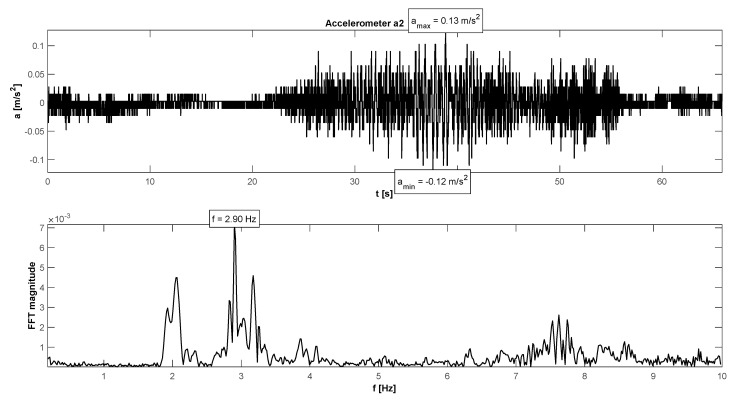
Signal of vertical vibrations in the time and frequency domain registered for 10 people walking.

**Figure 26:**
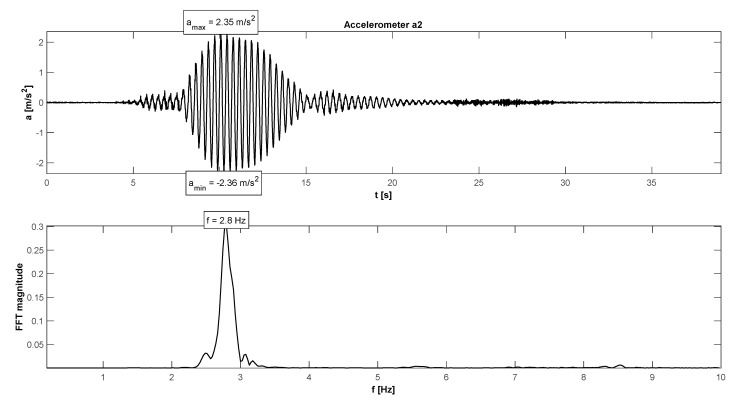
Signal of vertical vibrations in the time and frequency domain registered for 10 people running.

**Figure 27:**
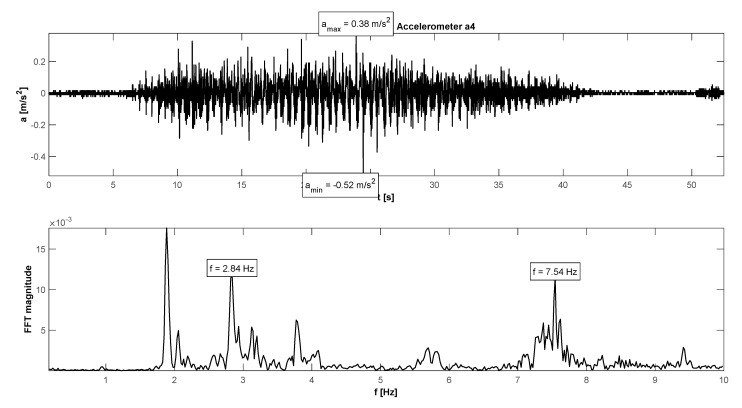
Signal of vertical vibrations in the time and frequency domain registered for the synchronized walking of 10 people.

**Figure 28:**
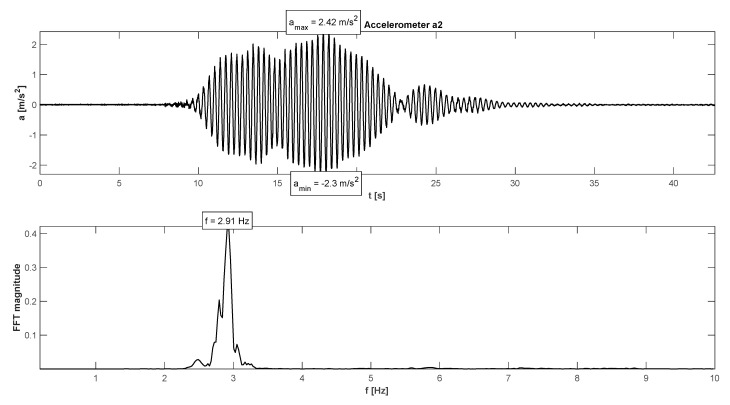
Signal of vertical vibrations in the time and frequency domain registered for the synchronized running of 10 people.

**Figure 29:**
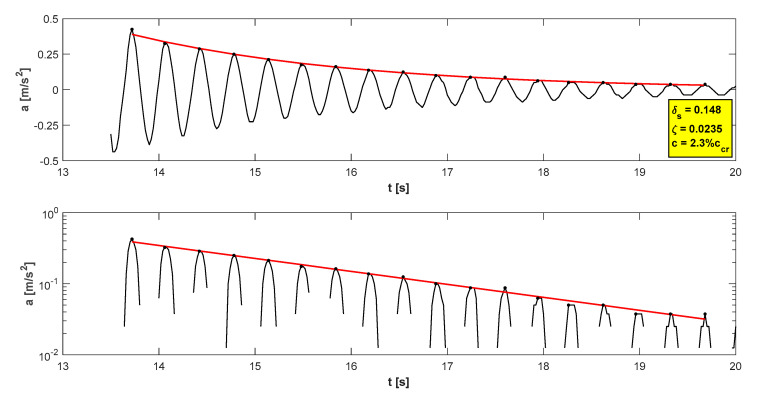
Determination of the damping coefficient.

The research programme has also proven proper behaviour of the footbridge in normal service conditions (see Table 6). The maximum accelerations were 0.13 m/s^2^ in walking conditions (Figure 25), 2.36 m/s^2^ in jogging conditions (Figure 26), and 1.04 m/s^2^ in fast running conditions. Synchronization of pedestrian activity caused higher dynamic response of the structure, e.g., up to 0.52 m/s^2^ in synchronized walking conditions (Figure 27), 2.42 m/s^2^ in synchronized jogging conditions (Figure 28), and 2.75 m/s^2^ at some vandal excitations such as half-crouching. The maximum comfort limit for vibrations was fulfilled in the case of walking and fast running. In the case of jogging, the minimum comfort limit was achieved (accelerations range limit: 2.20 m/s^2^ ÷ 3.30 m/s^2^ according to [30]). Human comfort was evaluated only for the normal activity of pedestrians. The damping ratio determined by using the Logarithmic Decrement Method was high and equals to 2.3% (Figure 29).

## 4. Discussion

The results of both research campaigns, conducted on glued-laminated wooden footbridges, are discussed below in relation to the identified frequencies (Table 7), the dynamic responses of the structures (Table 8,) and the damping coefficient (Table 9).

As it can be concluded from Table 7, when analysing the natural frequencies of both structures, the beam footbridge in Wrocław is much stiffer than the cable-stayed footbridge in the Pieniny mountains. However, the first flexural frequency (f_1_^V^ = 2.93 Hz) is within the critical frequency range for running pedestrians and is undesirable. In the case of the cable-stayed structure, the most critical frequency is connected with horizontal vibrations (f_1_^H^ = 1.10 Hz).

When comparing results presented in Table 8, both footbridges are not sensitive to vibrations induced by walking pedestrians. The low values of vertical accelerations are similar. However, in the case of people running, the acceleration values differ significantly—for the cable-stayed structure: a_max_^V^ = 1.11 m/s^2^ (comfort criteria fulfilled) and for the beam structure: a_max_^V^ = 2.36 m/s^2^ (comfort criteria exceeded). The dynamic response of the footbridge in Wrocław, which is twice the size, is connected with the first flexural frequency discussed above (2.93 Hz), which is close to the mean value of a step frequency for running pedestrians (2.70 Hz). It can be seen that sprinting does not cause a serious dynamic response in the case of both footbridges, which is a result of higher natural frequencies remaining outside the critical range for fast running. As a result of pedestrians’ movement synchronization, higher values of accelerations can be observed for the cable-stayed structure, which is connected with a much larger span of the footbridge (90 m vs. 40 m in the case of the beam footbridge), and consequently with lower stiffness and lower vibration resistance.

The values of damping (see Table 9) are in line with the values mentioned in the Eurocode EN 1991 [33] (in Part 1–4, Annex F.5). The value of logarithmic decrement of structural damping for timber bridges is defined in [33] as 0.06 ÷ 0.12. Other materials are characterized by much lower values of damping, e.g., for steel bridges δ_s_ = 0.02, and for composite and concrete bridges δ_s_ = 0.04 [33]. This means that timber is the structural material with the highest damping among materials listed in [33], and this positive feature makes the glued-laminated wood a very suitable option in terms of the vibration serviceability of footbridges.

## 5. Conclusions

The material used in the case of both analysed structures is modern, sustainable, and ecological. It is also user-friendly for pedestrians.

The choice of glued-laminated timber was accurate in terms of the architecture and the final aesthetic effect.

The technology of the material production enables easy shaping of structural elements, which is an additional positive design aspect.

Both tested footbridges differ from each other in structural systems and length of spans, but both are characterized by sufficient dynamic properties (especially in relation to the damping coefficient) and low sensitivity to vibrations.

High level of damping in footbridges made of glued-laminated wood is a very significant and crucial factor when determining the proper dynamic behaviour in a resonant zone of vibrations.

The structures discussed above are landmarks created with the application of unusual and innovative design solutions.

**Figure 30:**
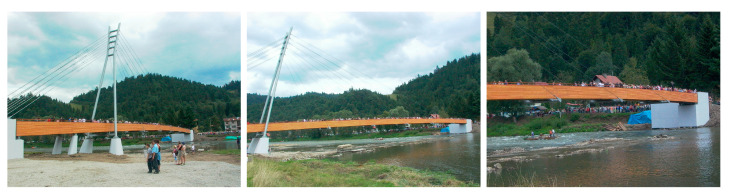
Snapshots of the crowd moving along the footbridge in the Pieniny mountains (direction Poland–Slovakia).

**Figure 31:**
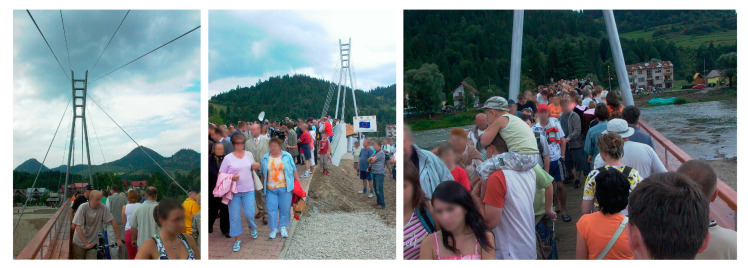
Very dense pedestrian traffic on the cable-stayed footbridge during the Opening Ceremony.

It should be stressed that the footbridge in the Pieniny mountains is one of the longest cable-stayed pedestrian bridges in the world. It is also the most recognizable Polish footbridge, which was found as one of the twenty most interesting objects built for tourism and recreation in Poland [34]. Until now, no claims to the dynamic behaviour have occurred, even in very crowded conditions (Figure 30 and Figure 31).

## Figures and Tables

**Table 1:** Calculated and identified frequencies of the deck’s vibrations.

No.	Results of Investigation f [Hz]	Results of Calculation f [Hz]	Type of Vibrations
1	1.22	1.35	1st vertical bending mode
2	2.16	2.47	2nd vertical bending mode
3	3.84	4.18	3rd vertical bending mode
4	1.10	1.41	1st horizontal bending mode

**Table 2:** Comparison of frequencies between the experiment and the updated FEM model.

No.	Results of Investigation Real Structure f [Hz]	Results of Calculation Updated FEM Model f [Hz]	Type of Vibrations
1	1.22	1.22	1st vertical bending mode
2	2.16	2.14	2nd vertical bending mode
3	3.84	3.41	3rd vertical bending mode
4	1.10	1.13	1st horizontal bending mode

**Table 3:** Comparison of calculation results.

No.	Original FEM Model		Updated FEM Model	
	f [Hz]	Type of Vibrations	f [Hz]	Type of Vibrations
1	1.35	1st vertical bending mode	1.13	1st horizontal bending mode
2	1.41	1st horizontal bending mode	1.22	1st vertical bending mode

**Table 4:** Values of extreme accelerations of the deck.

Test	No. of Pedestrians	a_max_^V^ [m/s^2^]	Comfort Criteria
Walking	12	0.21	Fulfilled
Running	12	1.11	Fulfilled
Fast running	12	1.38	Fulfilled
Synchronized walking	12	2.20	-
Synchronized running	12	3.14	-
Synchronized half-crouching	12	4.19	-

**Table 5:** Calculated and identified frequencies of the deck’s vibrations.

No.	Results of Investigation f [Hz]	Results of Calculation f [Hz]	Type of Vibrations
1	2.93	2.96	1st vertical bending mode
2	7.38	9.10	2nd vertical bending mode
3	2.63	1.31	1st horizontal bending mode
4	3.23	3.51	2nd horizontal bending mode

**Table 6:** Values of extreme accelerations of the deck.

Test	No. of Pedestrians	a_max_^V^ [m/s^2^]	Comfort Criteria
Walking	10	0.13	Fulfilled
Running	10	2.36	Exceeded
Fast running	10	1.04	Fulfilled
Synchronized walking	10	0.52	-
Synchronized running	10	2.42	-
Synchronized half-crouching	10	2.75	-

**Table 7:** Comparison of identified frequencies of the deck’s vibrations.

No.	Cable-Stayed Footbridge f [Hz]	Beam Footbridge f [Hz]	Type of Vibrations
1	1.22	2.93	1st vertical bending mode
2	2.16	7.38	2nd vertical bending mode
3	1.10	2.63	1st horizontal bending mode

**Table 8:** Comparison of extreme accelerations of the deck.

Test	Cable-Stayed Footbridge a_max_^V^ [m/s^2^]	Beam Footbridge a_max_^V^ [m/s^2^]
Walking	0.21	0.13
Running	1.11	2.36
Fast running	1.38	1.04
Synchronized walking	2.20	0.52
Synchronized running	3.14	2.42
Synchronized half-crouching	4.19	2.75

**Table 9:** Values of the damping coefficient.

Damping	Cable-Stayed Footbridge	Beam Footbridge
Damping coefficientc [%]	1.3c_cr_	2.3c_cr_
Logarithmic decrement of structural damping δ_s_ [−]	0.08	0.15

## Data Availability

The data presented in this study are available on request from the corresponding author.
